# Stronger compensatory thermal adaptation of soil microbial respiration with higher substrate availability

**DOI:** 10.1093/ismejo/wrae025

**Published:** 2024-02-12

**Authors:** Lingrui Qu, Chao Wang, Stefano Manzoni, Marina Dacal, Fernando T Maestre, Edith Bai

**Affiliations:** CAS Key Laboratory of Forest Ecology and Silviculture, Institute of Applied Ecology, Chinese Academy of Sciences, Shenyang, Liaoning, 110016, China; CAS Key Laboratory of Forest Ecology and Silviculture, Institute of Applied Ecology, Chinese Academy of Sciences, Shenyang, Liaoning, 110016, China; Key Laboratory of Terrestrial Ecosystem Carbon Neutrality, Institute of Applied Ecology, Chinese Academy of Sciences, Shenyang, Liaoning, 110016, China; Department of Physical Geography and Bolin Centre for Climate Research, Stockholm University, Stockholm, 10691, Sweden; Instituto Multidisciplinar para el Estudio del Medio ‘Ramón Margalef’, Universidad de Alicante, Alicante, 03690, Spain; Freie Universität Berlin, Institute of Biology, Berlin, 14195, Germany; Instituto Multidisciplinar para el Estudio del Medio ‘Ramón Margalef’, Universidad de Alicante, Alicante, 03690, Spain; Departamento de Ecología, Universidad de Alicante, Alicante, 03690, Spain; Key Laboratory of Geographical Processes and Ecological Security of Changbai Mountains, Ministry of Education, Northeast Normal University, Changchun, Jilin, 130024, China; Key Laboratory of Vegetation Ecology, Ministry of Education, Northeast Normal University, Changchun, Jilin, 130024, China

**Keywords:** microbial respiration, global warming, soil carbon decomposition, microbial thermal adaptation

## Abstract

Ongoing global warming is expected to augment soil respiration by increasing the microbial activity, driving self-reinforcing feedback to climate change. However, the compensatory thermal adaptation of soil microorganisms and substrate depletion may weaken the effects of rising temperature on soil respiration. To test this hypothesis, we collected soils along a large-scale forest transect in eastern China spanning a natural temperature gradient, and we incubated the soils at different temperatures with or without substrate addition. We combined the exponential thermal response function and a data-driven model to study the interaction effect of thermal adaptation and substrate availability on microbial respiration and compared our results to those from two additional continental and global independent datasets. Modeled results suggested that the effect of thermal adaptation on microbial respiration was greater in areas with higher mean annual temperatures, which is consistent with the compensatory response to warming. In addition, the effect of thermal adaptation on microbial respiration was greater under substrate addition than under substrate depletion, which was also true for the independent datasets reanalyzed using our approach. Our results indicate that thermal adaptation in warmer regions could exert a more pronounced negative impact on microbial respiration when the substrate availability is abundant. These findings improve the body of knowledge on how substrate availability influences the soil microbial community–temperature interactions, which could improve estimates of projected soil carbon losses to the atmosphere through respiration.

## Introduction

Global soils store at least twice as much carbon as that existing in the atmosphere [1]. Soil microbial respiration is one of the main processes of carbon loss from the soil to the atmosphere, releasing ~60 Pg C per year as carbon dioxide [2]. Temperature is one of the most important drivers of soil respiration in terrestrial ecosystems globally [3-5]. Warming-induced soil carbon loss via soil respiration has been estimated to increase by up to 190 Pg over the 21st century [6]. However, the direction and magnitude of the response of microbial respiration to ongoing global warming, in addition to the intricate underlying mechanisms, remain highly uncertain [7]. Short-term warming experiments have shown that the soil respiration rate increases with increasing temperature [8-11]. If this response persists in the long-term, it should result in self-reinforcing feedbacks to global warming [8, 12-15]. However, the results from long-term experiments have shown that the respiration rate may return to pretreatment levels after a few years of warming [11, 14, 16, 17]. This decline in microbial respiration rate in response to warming was considered as a compensatory response [18], indicating that soil carbon stocks are less vulnerable to the global climate change than currently feared.

Two possible mechanisms have been proposed to explain the compensatory response of soil microbial respiration to warming. The first mechanism is microbial thermal adaptation, which refers to the direct compensatory responses of microorganisms to warming across immediate to multigenerational time scales [17]. This adaptation is manifested by the physiological adjustment of individuals [16], evolutionary selection for genotypes within populations [19], and/or species turnover [20]. For example, a decrease in the conformational flexibility of enzymes and a reduction in cell membrane permeability following warming may impose constraints on the physiological process rates of individual microorganisms [21]. Moreover, an increase in the carbon use efficiency (CUE, the ratio of microbial growth to carbon uptake) of the microbial community has been proposed as a potential mechanism for the compensatory thermal response of microbial respiration to warming [22-24]. Alternatively, warming may also indirectly influence the supply and demand of resources by altering the quantity and quality of substrate available to microorganisms [25-27]. Several previous studies have shown that the enhanced response of microbial respiration to warming weakens due to substrate depletion [26, 28]. Additionally, microbial thermal adaptation implies that a reduction in microbial activity associated with the depletion of substrate after long-term warming [27]. Thus, substrate depletion might be an important factor influencing the thermal adaptation of soil microbial respiration [29]. Given that distinguishing between the effects of these two mechanisms is difficult [30], no consensus has emerged on how substrate availability affects the thermal adaptation of microorganisms [26, 27].

Several attempts have been made to examine the impacts of substrate depletion and/or microbial thermal adaptation to warming on soil microbial respiration [29]. For example, in short-term laboratory incubation studies, microbial respiration rates were measured using soils collected from multiple ecosystems across the USA [18] and from global drylands [31] under sufficient substrate availability. They found that microbial respiration rates increased less at higher incubation temperatures than at lower incubation temperatures. But microbial respiration rates were lower in soils from warmer regions than in those from colder regions under the same incubation temperature, suggesting the influence of thermal adaptation. By contrast, some studies on soils from alpine meadows and temperate forests have shown that the compensatory temperature response to microbial respiration is regulated by substrate availability rather than by thermal adaptation [26, 28]. In addition, the results from a long-term warming experiment in an alpine meadow showed that changes in microbial respiration rates were regulated by the combined effects of a reduction in soil carbon pools (i.e. substrate depletion) and thermal adaptation [29]. Therefore, the role of substrate availability in thermal adaptation appears to be inconsistent among these studies. It is important to consider the interplay of microbial physiological processes with substrate availability when addressing the relationship between soil microbial respiration and temperature.

To assess the effects of thermal adaptation and substrate availability on soil microbial respiration rates, we collected soils from 11 sites along a north–south forest transect (mean annual temperature (MAT), ranging from −4.4°C to 22.4°C) in eastern China, and we incubated the soils at three temperatures (i.e. 12°C, 20°C, and 28°C) with or without substrate addition. The microbial respiration rates were fitted to an exponential temperature response curve based on a widely used empirical model [32]. Linear mixed-effect models were then used to assess the effects of thermal adaptation and substrate availability on microbial respiration rates in both short-term (i.e. incubation) and long-term (MAT) temperature gradients while standardizing the effects of important drivers of soil microbial respiration (i.e. microbial biomass and soil properties). The results from this survey were compared to two additional independent datasets that share a similar experimental design [18, 31].

**Figure 1:**
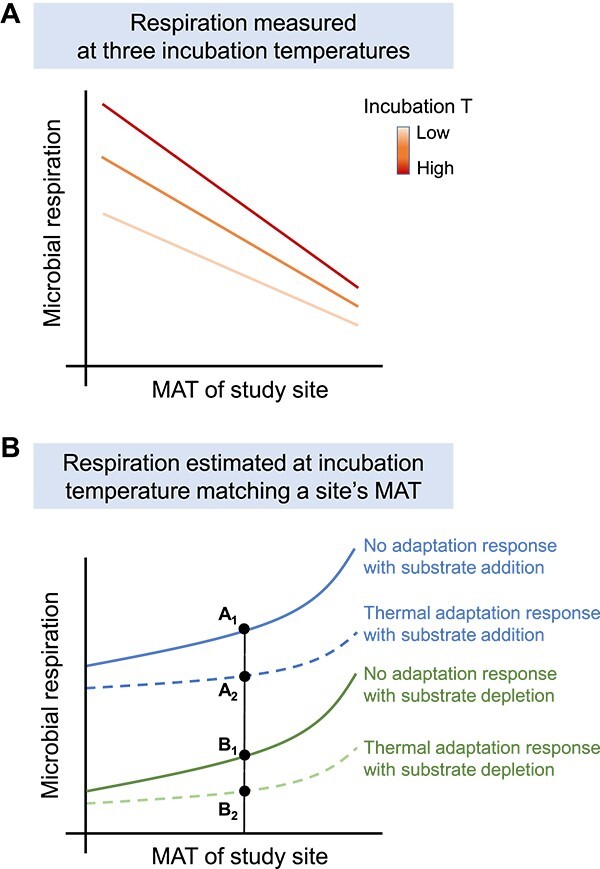
Conceptual paradigm depicting the effect of substrate availability on microbial thermal adaptation; (a) compensatory thermal adaptation of microbial respiration rate to climate warming, that is respiration rate at the mean microbial biomass across all sites decreases with increasing MAT; we hypothesized that microbial respiration rate was higher in soils from colder environments than those from warmer environments when incubated at the same incubation temperature (*T*); further, the differences in respiration rates between incubation temperatures should be larger for samples from colder environments than for those from warmer environments because thermal adaptation effects should be greater in warmer regions; (b) microbial respiration rate increases with increasing MAT during incubation at *T* = MAT under excess substrate addition (*A*_1_ and *A*_2_) and substrate depletion (*B*_1_ and *B*_2_); microbial respiration rate is higher when substrate is added than without it (e.g. *B*_1_ < *A*_1_ or *B*_2_ < *A*_2_) due to substrate depletion effect; thus, the substrate depletion effect without thermal adaptation is calculated as *A*_1_ − *B*_1_ at the same *T*; accordingly, the substrate depletion effect with thermal adaptation is calculated as *A*_2_ − *B*_2_; microbial respiration rate is lower with microbial thermal adaptation than without adaptation due to the compensatory response (e.g. *A*_2_ < *A*_1_ or *B*_2_ < *B*_1_); thus, the microbial thermal adaptation effect with substrate addition is calculated as *A*_1_ − *A*_2_ at the same *T*; accordingly, the microbial thermal adaptation effect with substrate depletion is calculated as *B*_1_ − *B*_2_; the overall difference in microbial respiration *A*_1_ − *B*_2_ is because of either the microbial thermal adaptation and the interaction of microbial thermal adaptation with substrate depletion, or substrate depletion and interaction of substrate depletion with microbial thermal adaptation.

We hypothesized that the soil microbial respiration rates normalized by the mean microbial biomass across all sites decrease with increasing MAT (Fig. 1A) and that the differences of respiration rates between incubation temperatures (e.g. 28°C vs. 20°C and 20°C vs. 12°C) are lower in warmer sites than in those from colder ones due to microbial thermal adaptation. We also hypothesized that the magnitude of the effect of thermal adaptation on soil microbial respiration could be reduced by substrate depletion (Fig. 1B) because the low substrate availability often suppresses the production and/or activities of microbial enzymes that are involved in thermal adaptation.

## Materials and methods

### Study sites and field survey

Soil samples were collected from 11 forest sites along a north–south transect in eastern China in July 2019. These sites covered three forest types: temperate forests (MH, Mohe; DXAL, Daxinganling; WY, Wuying; CBS, Changbai Mountain; DLS, Dongling Mountain), subtropical forests (BTM, Baotianman; JGS, Jigong Mountain; WYS, Wuyi Mountain; DHS, Dinghu Mountain), and tropical forests (JFL, Jianfengling; XSBN, Xishuangbanna). The MAT of sites ranged from −4.4°C to 22.4°C. More detailed information of sampling sites can be found in the supplementary material (Supplementary Table S1).

At each site, five 20 × 20 m plots were set up within a 500 × 500 m area. Fifty soil cores (2.5 cm in diameter) within 0–10 cm were taken from each plot and were then mixed thoroughly to generate one composite sample per plot, resulting in a total of 55 soil samples (5 replicates per site × 11 sites). Fresh soil samples were sieved through a 2-mm sieve to remove all visible roots and litter fragments. We stored soil samples at 4°C for <3 days before the experiment began. A previous study showed that soil respiration and its temperature sensitivity are not significantly affected by sieving and storing at 4°C for <7 weeks [33]. Thus, the soil pretreatment approach had limited impact on the following microbial respiration rate analyses [4, 10].

### Measuring microbial respiration rate

We measured microbial respiration rate after a short-term incubation (24 h) to prevent microbial adaptation to the incubation temperatures used [4, 10]. To separate the effects of thermal adaptation and substrate depletion on microbial respiration, we assessed microbial respiration rates both with sufficient substrate addition (to test for thermal adaptation alone) and without substrate addition (to test for both thermal adaptation and substrate depletion). The most common labile substrates available to soil microorganisms are sugars, amino acids, and organic acids [34]. Empirical evidence from Bradford *et al*. [18] suggests that soil microbial respiration has the similar compensatory thermal responses to the addition of excess glucose, glycine, and oxalic acid. Thus, 2 ml of 75 mM glucose (i.e. a dose of 10.8 mg C g^−1^ dry soil) was chosen to exceed microbial demand across the present assay. Briefly, three fresh soil subsamples (20 g) were taken from each fresh sample. All subsamples were adjusted to 55% water-holding capacity (WHC) for optimal microbial activity and were preincubated under three incubation temperatures (i.e. 12°C, 20°C, and 28°C) for 24 h to avoid distorting the signal of microbial respiration. Given that the soils varied substantially in terms of their moisture during the field collection, fixing the soil moisture to the same percentage of the WHC facilitated the comparison of microbial processes among multiple sites [35, 36]. These incubation temperatures covered the optimal range for metabolic activity of both cold- and warm-adapted microorganisms [18]. Following preincubation, two aliquots of 1 g of each preincubated soil were weighted into 20-ml vials, respectively. Then, 2 ml of 75 mM glucose solution was added to the substrate addition treatment to remove the “substrate depletion” effect. Subsequently, the vials were capped and flushed with CO_2_-free air (22% O_2_ + 78% N_2_) until the initial CO_2_ concentration decreased to 0 ppm. This process generally took less than 2 min. Then, a needle was be inserted into the rubber pad of the vial cap, the excess gas was released, and then we immediately pulled out the needle. This ensured that the air pressure in the vials is at normal levels. In addition, three empty vials were set as negative controls for CO_2_ concentration analysis following the same procedure. All samples were incubated for 24 h under their corresponding preincubation temperatures (i.e. 12°C, 20°C, and 28°C). After incubation, the air pressure in the head of incubation vials was monitored using an air gage (PG-100-102GP, Copal Electronics, Japan), which would be used as an input parameter in the following soil respiration calculation (Equation (1)). Subsequently, 10 ml gas in the headspace of each vial was collected with a syringe, and the CO_2_ concentration was determined immediately by a gas chromatograph system (GC-7890B, Agilent Technologies, USA). We tested 330 samples in total (55 soil samples × 3 incubation temperatures × 2 treatments). The soil microbial respiration rate (*R_m_*, μg C g soil^−1^ h^−1^) was calculated according to the following equation:


(1) \begin{equation*} {R}_m=\frac{\left[\text{C}{\text{O}}_2\right]}{\text{DM}\times t}\times \frac{p\times n}{R_{\text{gas}}\times T}\times V \end{equation*}

where [CO_2_] is the CO_2_ concentration accumulated during the 24-h incubation period as a result of microbial respiration (ppm), DM is soil dry mass (g), *t* is the incubation time (hours), *p* is the headspace air pressure within vial during incubation (kPa), *n* is the molecular mass of the element C (12.01 g mol^−1^), and *R*_gas_ is the ideal gas constant (8.314 J mol^−1^ K^−1^), *T* is the absolute temperature during the incubation (K), and *V* is the headspace volume of the incubation vial (l).

### Soil properties and microbial biomass

Soil WHC was measured by repeatedly saturating soils (20 g fresh soil) with deionized water for 2 h, draining in a funnel with an ash-free cellulose filter paper for 8 h, and then drying in 105°C for 24 h. Soil pH was measured at a 1:2.5 ratio of air-dried soil to deionized water by a pH electrode (Leici, Shanghai, China). Soil organic carbon (SOC) and total nitrogen (TN) were measured using an elemental analyzer (MAT-253, Thermo Fisher Scientific, USA) with ball-milled dry soil. Soil clay content (Clay) was measured by the hydrometer method [37].

We used the chloroform fumigation extraction (CFE) method to estimate the soil microbial biomass [38]. Briefly, 20 g of fresh samples were weighed in a 100-ml beaker, placed in a desiccator, and fumigated in the dark with alcohol-free chloroform for 48 h. Dissolved organic carbon (DOC) and nitrogen (DON) were extracted using 0.5 M K_2_SO_4_ from fumigated and nonfumigated soils. The difference in DOC between the fumigated and nonfumigated soils was considered as the microbial biomass carbon with a conversion coefficient of 0.45.

### Empirical relationship between microbial respiration rate and temperature

In the subsequent analysis, we presented a data-driven model to link the respiration rate (calculated from Equation (1)) to incubation temperatures, MAT, and other soil variables. The relationship between microbial respiration rate (*R_m_*) and incubation temperatures could be well described by the exponential response function [32]:


(2) \begin{equation*} {\text{R}}_{\text{m}}=\alpha \times{\text{e}}^{\beta \text{T}} \end{equation*}

where *R_m_* is the microbial respiration rate, *T* is the soil temperature during the incubation, *α* is the basal respiration rate, and *β* represents the sensitivity to change in temperature, which is related to the commonly reported *Q*_10_ value. Then, a natural-log-transformation was applied to Equation (2):


(3) \begin{equation*} \ln \kern0.33em \left({\text{R}}_{\text{m}}\right)=\ln \left(\alpha \right)+\beta \times \text{T} \end{equation*}

Subsequently, we built a linear model between ln(*α*) and its key influencing variables (MAT, microbial biomass, SOC, pH, Clay, TN, and soil DOC:DON) [10, 39-42]:


(4) \begin{eqnarray*} \ln \left(\alpha \right)=&\!\!\!\!\! a\times \text{MAT}+\kern0.33em b\times \text{Biomass}+\kern0.33em c\times \text{SOC}+d\times \text{pH}\nonumber\\&\ +\,e\times \text{Clay}+\kern0.33em f\times \text{TN}+\kern0.33em g\times \text{DOC}:\text{DON} \end{eqnarray*}

Assuming that the temperature sensitivity parameter *β* is linearly related to MAT [8], we built the model of *β*:


(5) \begin{equation*} \beta =h\times \text{MAT}+\kern0.33em i \end{equation*}

Inserting Equations (4) and (5) into Equation (3), a linear model, including an interaction term between MAT and T, was obtained:


(6) \begin{align*} \ln \left({\text{R}}_{\text{m}}\right)=&\ a\times \text{MAT}+\kern0.33em b\times \text{Biomass}+\kern0.33em c\times \text{SOC}+d\times \text{pH}\nonumber\\&+e\times \text{Clay}+\kern0.33em f\times \text{T}\text{N}+\kern0.33em g\times \text{DOC}:\text{DON}\nonumber\\&+\kern0.33em h\times \text{MAT}\times \text{T}+\kern0.58em i\times \text{T} \end{align*}

We used a linear mixed-effect model to estimate the coefficients in Equation (6). We considered the sampling location as a random variable. In addition, variance inflation factors (<10) were used to exclude variables to avoid multicollinearity [43]. We removed soil TN in the final model due to its high multicollinearity. Subsequently, we removed SOC because it was highly correlated with the microbial biomass (*R*^2^ > 0.7, Supplementary Tables S2 and S3). We also removed soil pH and soil DOC:DON because they had limited effects on the microbial respiration rate in the studied soils (Supplementary Tables S2 and S3). Thus far, we have successfully obtained the best-performing model after iterative data fitting, as presented in Supplementary Tables S2–S5, which show the results of the model selection process. Linear mixed-effect models were fitted with a Gaussian error distribution using the “lmer” function in the lme4 package [44].

We also analyzed the relationship between the microbial respiration rate and temperature using the macromolecular rate theory (MMRT) model and square-root model (Supplementary Tables S6 and S7). Both models had weaker prediction power for our dataset than the exponential response model (Supplementary Table S8), and thus their results were not presented in the main text. All models were constructed in R statistical software in version 4.1.3 [45].

### Assessing the effects of substrate availability and thermal adaptation on respiration

We used the unstandardized coefficients obtained from the best-performing model, shown in Equation (6) (Table 1), to estimate the potential respiration rates for each soil at an incubation temperature (e.g. 12°C, 20°C, and 28°C). In order to test the effect of thermal adaptation on respiration, we set the incubation temperature, in Equation (6), to be the same as the MAT of each sampling site, while keeping all other variables (i.e. microbial biomass and soil texture) constant at the mean value across all sites. By contrast, to calculate the soil respiration without adaptation effect, the MAT parameter was removed from the model by setting its coefficient to zero (*a* = *h* = 0) while keeping other coefficients identical. The models were also run for treatments under substrate addition to test if the substrate could affect the thermal adaptation of respiration.

**Table 1:** Parameters of models used to assess microbial thermal adaptation.

Variables		Intercept	*T*	MAT	Biomass	Texture	MAT × *T*	Marginal *R*^2^	Conditional *R*^2^	AIC
This study	No substrate addition	**−3.237 ± 0.244**	**0.077 ± 0.005**	*−0.026 ± 0.013*	**0.452 ± 0.045**	**0.013 ± 0.003**	−0.0002 ± 0.0004	0.83	0.92	156.84
	Substrate addition	**−1.354 ± 0.184**	**0.058 ± 0.005**	**−0.029 ± 0.009**	**0.275 ± 0.032**	*0.005 ± 0.002*	0.0005 ± 0.0003	0.80	0.85	127.14
Bradford *et al*. (2019)	No substrate addition	**−2.383 ± 0.247**	**0.052 ± 0.008**	*−0.027 ± 0.016*	**0.005 ± 0.001**	**−0.005 ± 0.003**	0.00002 ± 0.00064	0.58	0.83	286.55
	Substrate addition	**−1.461 ± 0.235**	**0.056 ± 0.005**	**−0.042 ± 0.013**	**0.005 ± 0.001**	*−0.005 ± 0.003*	0.0005 ± 0.0004	0.65	0.95	174.97
Dacal *et al*. (2019)	No substrate addition	**1.617 ± 0.136**	**0.048 ± 0.004**	**−0.017 ± 0.006**	0.062 ± 0.093	**−0.011 ± 0.001**	**−0.0005 ± 0.0002**	0.50	0.78	558.14
	Substrate addition	**2.160 ± 0.141**	**0.046 ± 0.004**	**−0.031 ± 0.006**	0.136 ± 0.098	**−0.015 ± 0.001**	0.0002 ± 0.0002	0.57	0.83	572.54

**Figure 2:**
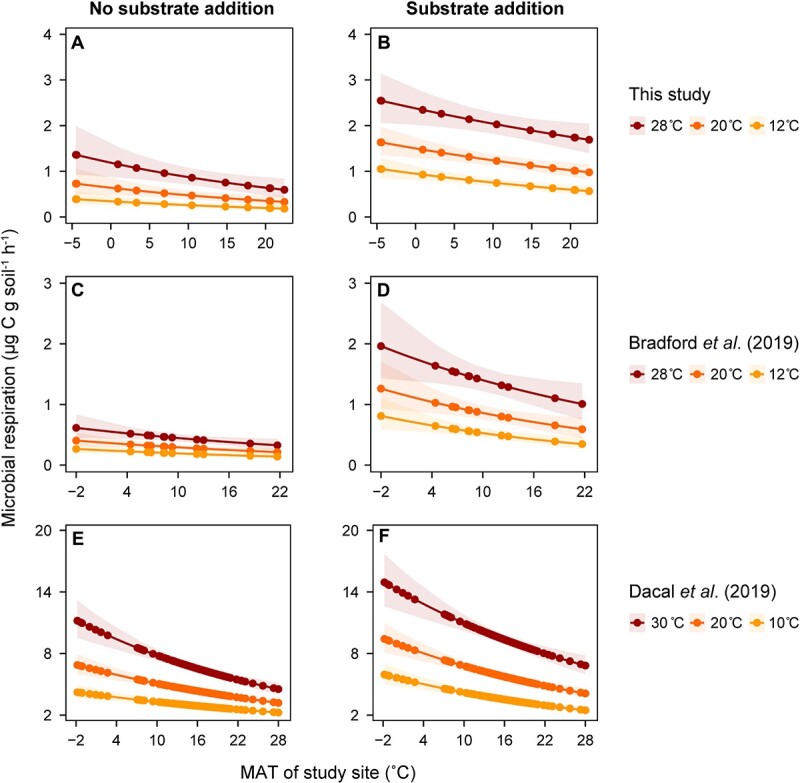
Model estimated effects of MAT on microbial respiration without and with substrate addition; (a) and (b) for the dataset from this study; (c) and (d) for the dataset from Bradford *et al*. [18]; (e) and (f) for the dataset from Dacal *et al*. [31]; microbial respiration rates (points) were estimated using the unstandardized coefficients from models given in Table 1, by increasing MAT and incubation temperature values in the regression equation systematically from the lowest to highest values observed in each study, and the values of the other variables (microbial biomass and soil texture) were held as the averages across all sites; the shaded areas show the standard deviation of potential soil microbial respiration rates at each incubation temperature (*T*).

**Figure 3:**
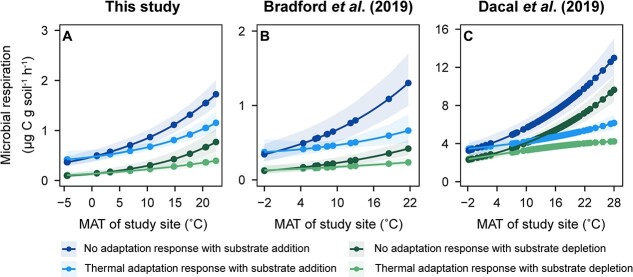
Estimated effects of microbial thermal adaptation and substrate availability on microbial respiration; (a) for the dataset from this study; (b) for the dataset from Bradford *et al*. [18]; (c) for the dataset from Dacal *et al*. [31]; microbial respiration rates (points) were calculated using the unstandardized coefficients from models given in Table 1; for the scenario with thermal adaptation, incubation temperature (*T*) was set to be the same as MAT, while the other variables (microbial biomass and soil texture) were held at the averages across all sites; by contrast, in the scenario without thermal adaptation, the coefficient of MAT was set to zero; the calculation was conducted for soil incubation with and without substrate addition.

Unstandardized coefficients (mean ± standard deviation) and *R*^2^ values for the best-performing linear mixed-effect models shown in Equation (6) by using data from this study, Bradford *et al*. [18], and Dacal *et al*. [31]. The effect of MAT on soil microbial respiration rates were assessed with or without substrate addition. Microbial respiration rates were ln-transformed to meet normality assumptions. Unstandardized coefficients were used to plot Figs 2 and 3, where predicted values were back-transformed from ln-predicted respiration rates before plotting. The negative effect of MAT and the self-reinforcing effect of incubation temperature (*T*) are consistent with our hypothesis shown in Fig. 1. Significant (*P* < .05) and marginally significant (.05 < *P* < .1) coefficients are shown in bold and italics, respectively. Marginal and conditional *R*^2^ values were calculated using a method that retains the random effect structure.

The effects of thermal adaptation and substrate depletion on microbial respiration can be quantified as follows (Fig. 1B):


(7) \begin{equation*} \delta ={A}_1-{A}_2, \end{equation*}

(8) \begin{equation*} \varepsilon ={A}_2-{B}_2, \end{equation*}

(9) \begin{equation*} \theta ={A}_1-{B}_1, \end{equation*}

(10) \begin{equation*} \vartheta ={B}_1-{B}_2, \end{equation*}

where *A*_1_ and *A*_2_ represent the microbial respiration rates without and with adaptation response in the substrate addition treatment, respectively; *B*_1_ and *B*_2_ represent the microbial respiration rates without and with adaptation response in the substrate depletion treatment, respectively; $\delta$ is the microbial thermal adaptation effect on respiration with substrate addition; $\varepsilon$ is the substrate depletion effect on respiration with thermal adaptation; $\theta$ is the substrate depletion effect on respiration without thermal adaptation; and $\vartheta$ is the microbial thermal adaptation effect on respiration with substrate depletion.

Finally, the overall compensatory thermal response of microbial respiration ($\varphi$) was calculated as:


(11) \begin{equation*} \varphi ={A}_1-{B}_2=\delta +\varepsilon =\theta +\vartheta . \end{equation*}

As shown in Fig. 1B, we can now quantify the relative magnitude of microbial thermal adaptation and substrate depletion to the overall thermal response $\varphi$:

- thermal adaptation effect with substrate addition:

(12) \begin{equation*} \rho =\frac{\delta }{\varphi }=\frac{A_1-{A}_2}{A_1-{B}_2}, \end{equation*}

- substrate depletion effect with adaptation:

(13) \begin{equation*} \sigma =\frac{\varepsilon }{\varphi }=\frac{A_2-{B}_2}{A_1-{B}_2}, \end{equation*}

- substrate depletion effect without adaptation:

(14) \begin{equation*} \tau =\frac{\theta }{\varphi }=\frac{A_1-{B}_1}{A_1-{B}_2}, \end{equation*}

- thermal adaptation effect with substrate depletion:

(15) \begin{equation*} \omega =\frac{\vartheta }{\varphi }=\frac{B_1-{B}_2}{A_1-{B}_2}. \end{equation*}

To verify the generality of our predictive model, we applied it to two independent large-scale datasets from previous studies [18, 31]. In these two datasets, the microbial respiration rates were all measured after a short-term incubation to prevent thermal adaptation to incubation temperatures (4 and 10 h for [18] and [31], respectively) using gas chromatography mentioned in Bradford *et al*. [18] and MicroResp approach mentioned in Dacal *et al*. [31], respectively. In addition, the microbial biomass was estimated using the phospholipid fatty acid and CFE methods mentioned in these studies. Sand content was included in the models for Bradford *et al*. [18] and Dacal *et al*. [31], but it was not used in our dataset, in which we used soil clay content instead. We were able to compare the coefficients obtained by fitting Equation (6) to datasets from these studies and in ours because no interaction was included between the temperature and texture or microbial biomass in the mixed-effect models.

## Results

### Relationships between measured soil microbial respiration rate and temperature

Soil microbial respiration rates increased significantly with increasing incubation temperatures across the forest soils studied, with respiration rates at 28°C being two to three times greater than those at 12°C (Fig. 2). Additionally, substrate addition accelerated the microbial respiration rates in all the soils (Supplementary Fig. S1).

We used the exponential thermal response function (Equation (2)) and linear mixed modeling (Equation (6)) to simulate the effect of temperature on soil respiration while considering other important factors, such as incubation temperatures, MAT, soil texture, and microbial biomass (Table 1). The predictive models performed well for both our data and those from Bradford *et al*. [18] and Dacal *et al*. [31], with the overall *R*^2^ ranging from 0.50 to 0.83 across all studies and treatments (Table 1).

The models showed that the soil microbial respiration rates obtained under the same incubation temperature were negatively correlated with the corresponding MATs (Fig. 2) regardless of the incubation temperatures or the treatments applied in most cases. However, the unstandardized coefficients for MAT in our dataset and that in the Bradford *et al*. [18] dataset were marginally significant (*P* = .060 and .096, respectively; Table 1). The absolute value of the slope of the linear relationship between MAT and respiration increased with increasing incubation temperatures (Fig. 2). This result is consistent with the lower increase in respiration rates with warming for microorganisms adapted to higher temperatures than for those adapted to lower temperatures. Microbial biomass, incubation temperature, and MAT were the most important variables influencing microbial respiration in the three datasets evaluated, regardless of treatments, while the soil texture influenced soil respiration more than microbial biomass in dryland ecosystems (Supplementary Tables S4, S5, and S9).

### Modeled thermal adaptation of respiration and interactions with substrate availability

We set the model incubation *T* equal to the MAT of each site or MAT coefficients to zero to assess the effects of thermal adaptation on microbial respiration. We found that modeled microbial respiration rates increased with increasing MAT, but this trend was dampened by thermal adaptation (Fig. 3). The dampening effects were greater with increasing MAT, which is consistent with the hypothesis of a compensatory response.

**Figure 4:**
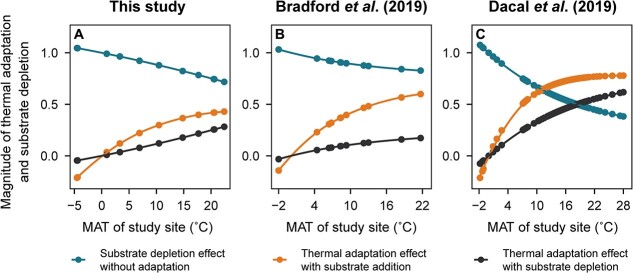
Relative magnitude of microbial thermal adaptation and substrate depletion on microbial respiration; (a) for the dataset from this study; (b) for the dataset from Bradford *et al*. [18]; (c) for the dataset from Dacal *et al*. [31]; the point values were calculated using data from Fig. 3 and Equations (12)–(15) given in the Materials and methods; higher values indicate the stronger influence on the overall compensatory response of microbial respiration.

Based on the conceptual results (Fig. 1B) and the estimated respiration rates with or without substrate addition in Fig. 3, we evaluated the relative magnitude of microbial thermal adaptation with or without substrate addition using Equations (12)–(15). We found that the effect of thermal adaptation on microbial respiration increased with MAT in the three datasets (Fig. 4). Such an effect increased more under an excess substrate (yellow vs. black line, Fig. 4). Additionally, the effects of substrate availability on microbial thermal adaptation were most pronounced at sites with MAT between 10°C and 20°C while tapering off in warmer climates.

## Discussion

In this study, we provide empirical evidence across multiple ecosystems that microbial thermal adaptation is more relevant in warmer areas, especially when the substrate is not a limiting factor. To quantify the magnitude of thermal adaptation of soil microbial respiration, previous studies have measured temperature sensitivity using the Arrhenius function, the *Q*_10_ model, the MMRT, and empirical models (e.g. the square-root model and Lloyd function) [32, 46-48]. Moreover, linear mixed-effect models and structural equation models have been used to investigate which factors (e.g. temperature and substrate availability) affect microbial respiration [18, 29, 31]. In this study, we built a predictive model based on an empirical exponential response function [32] in which the temperature sensitivity and basal respiration rates depend on the climatic and soil conditions of the sampling sites. Our model predicted that soil microbial respiration rates are consistently driven by pedoclimatic conditions at the sampling sites and by incubation temperatures both in our dataset from a forest transect in China and in two previously published datasets from different ecosystems [18, 31]. The fixed effects in our model captured a large part of the variation in soil microbial respiration, indicating that the selected independent variables indeed have a high explanatory power. Our findings thus provide empirical evidence that thermal adaptation is a mechanism for reducing the temperature sensitivity of soil microbial respiration, possibly weakening the self-reinforcing carbon cycle–climate feedback associated with global warming. In addition, our microbial respiration model could complement the temperature response function in most current ecosystem models that only account for current temperature conditions and neglect the microbial thermal adaptation [49]. However, we also caution that our model should be tested with additional datasets from ecosystem types that were not considered in this study.

Our results indicate that incubation temperatures, MATs, soil texture, and microbial biomass are important drivers of soil respiration (Table 1). These results are in agreement with previous findings [11, 17, 31, 50]. However, we did not consider the indirect effects of temperature on respiration, such as those mediated by soil moisture. In fact, warming may decrease the soil water availability, which can impact the thermal response of microbial activity and biomass [51]. Therefore, a low water availability in the soil might indirectly amplify the compensatory thermal response of microbial respiration, reducing the microbial activity. In fact, soil drying decreases the substrate diffusion rates to microorganisms [52] and inhibits the enzymatic activities necessary to breakdown complex polymers to monomers usable by microorganisms [53], leading to a suppression of the response of soil microbial respiration to warming.

Substrate availability controlled the microbial respiration and its response to increasing temperature (Supplementary Fig. S1) because soil microorganisms tend to be carbon-limited [54]. Thus, substrate availability could also affect the temperature sensitivity of microbial activity [26, 27] and should be considered when studying the thermal adaptation of soil microbial respiration. Here, we measured and modeled the soil microbial respiration rates under optimal moisture conditions (i.e. 55% WHC), which balanced the substrate availability and effective soil aeration; therefore, we did not examine the effect of limited soil water availability. However, in future studies, it will be essential to evaluate how reduced soil moisture levels affect the quality and quantity of the available substrate and how they interact with soil microbial thermal adaptation.

Consistent with our first hypothesis, we found that under the same incubation temperature, microbial respiration rates were lower in warmer sites than colder ones regardless of substrate availability (Fig. 2). These results were most likely driven by microbial thermal adaptation, which has been reported in many previous studies [4, 16, 55, 56]. The increase in MAT at the sampling sites represented a long-term warming effect because other soil factors were constrained in the model. Warm-adapted microorganisms exhibited a lower increase in respiration rates than cold-adapted microorganisms with increasing incubation temperatures (Fig. 2), as predicted by the conceptual model (Fig. 1A). These findings further support the occurrence of compensatory responses of soil respiration to increasing temperatures.

We found that the effect of microbial thermal adaptation was negative when the MAT was <0°C (Fig. 4), indicating an enhanced soil microbial respiration response under increasing temperatures in these areas with MAT <0°C, which contrasts with the compensatory thermal response found in regions with MAT >0°C. Microorganisms in frozen soils (i.e. at temperatures <0°C) could be alive, but they would be mostly inactive [57] due to the limited availability of liquid water and low temperature. However, as temperatures rise, the soil environment undergoes a transformation, becoming more conducive to microbial activity due to a variety of processes. For example, the frozen water in the soil melts, which improves the transport of water, enzymes, and nutrients within the soil matrix, consequently stimulating the reactivation of dominant microorganisms and inducing an increase in the soil respiration rates [58]. These results suggest that the magnitude of self-reinforcing carbon cycle-climate feedback is greater in areas with a mean MAT <0°C than in areas with mean MAT >0°C, highlighting the extreme importance of these areas in global warming studies. However, we only had two sites in the Chinese forest transect with MAT <0°C; therefore, this conclusion needs to be substantiated by additional studies conducted in regions with MAT <0°C.

The magnitude of microbial thermal adaptation to the compensatory response of respiration to warming increased with increasing MAT (Fig. 4), indicating greater effects of thermal adaptation on microbial respiration in warmer soils. The effect of thermal adaptation on microbial respiration was generally greater under substrate addition than under substrate depletion (Fig. 4), which agrees with our second hypothesis. These observed patterns could be caused by the decrease in catalytic rates of enzymatic reactions, the diminished conformational flexibility of enzymes, and the weakened permeability of cell membranes with the prolonged duration of warming [59, 60]. These declines in enzymatic activities could cause stronger effects on soil respiration when substrate availability is greater, resulting in greater thermal adaptation.

The estimated relative effect of substrate depletion without thermal adaptation on soil respiration decreased with increasing MAT (Fig. 4), indicating that microorganisms were less limited by substrate availability in warmer areas than colder ones. Substrate availability is mainly controlled by the activity of enzymes and the physicochemical protection of soil organic matter on mineral surfaces and within aggregates [61]. Enzymatic activities are generally lower in colder areas than in warmer regions, which means that less labile substrates are available for soil microorganisms in colder areas for a given carbon input to the soil. Comparing with warmer areas, molecules in reactions with high activation energies decompose slowly in colder areas but might become more available under increasing temperatures [62, 63]. In addition, carbon protected by the soil matrix is not available to microorganisms [64], and warming could increase the rates of substrate release to microorganisms from the organo-mineral surfaces [62]. These findings suggest that warming could affect soil respiration not only via kinetic changes in metabolic rates but also via substrate availability. In addition, shifts in the microbial community composition can also play a role in regulating the compensatory response of soil microorganisms to increasing temperatures. The microbial community composition changes with long-term warming (e.g. increasing thermophilic taxa), resulting in shifts in the dominant strategy and thus thermal adaptation at the whole-community level [20]. For example, slow-growing microorganisms that use recalcitrant carbon sources more efficiently (i.e. K-strategy-dominant microbial community) are usually better adapted to the changing climate than fast-growing microorganisms thriving in soils enriched with labile carbon (i.e. r-strategy-dominant microbial community) [65]. Previous studies have shown that microbial K-strategists have higher CUE than microbial r-strategists [66]. Thus, warming might select for soil microbial community dominated by K-strategists with higher CUE, thereby weakening the thermal response of soil respiration. In contrast to these arguments, CUE estimated via data assimilation appears to decrease with MAT [67]. These inconsistencies suggest that despite our understanding of substrate and adaptation effects on microbial respiration, we still do not fully understand how temperature modulates microbial growth and thus long-term C storage.

## Conclusion

We developed a predictive model to investigate the thermal adaptation of soil microbial respiration under different substrate availability conditions across regional and global scales. Utilizing this model, we demonstrated that the negative effects of thermal adaptation on soil microbial respiration are more pronounced in warmer regions, and these effects escalate with an increase in substrate availability. Our findings demonstrated that increased temperature/substrate availability in soil enhances the effect of thermal adaptation on soil microbial respiration. These findings will contribute to a better understanding of the mechanisms underlying microbial responses to warming and could contribute to parameterizations of soil carbon cycling models that include adaptation effects.

## Author contributions

C.W. and E.B. designed the original idea of the study. C.W. and L.Q. developed the field study and conducted all field operations and laboratory work. L.Q., C.W., S.M., and E.B. performed the data analyses. The paper was written by L.Q., C.W., S.M., and E.B. with the contributions from all coauthors.

## Conflicts of interest

The authors declare that they have no known competing financial interests or personal relationships that could have appeared to influence the work reported in this paper.

## Funding

This work was financially supported by National Key Research and Development Program of China (Grant No. 2023YFE012400), Key Research Program of Frontier Sciences (Grant No. ZDBS-LY-DQC019), National Natural Science Foundation of China (Grant Nos. 32371845 and 42322306), Major Program of Institute of Applied Ecology, Chinese Academy of Sciences (Grant No. IAEMP202201), International Partnership Program of Chinese Academy of Sciences (Grant No. 064GJHZ2022054FN), and the Youth Innovation Promotion Association CAS to C.W. (Grant No. Y2022064). S.M. has received funding from the European Research Council under the European Union’s Horizon 2020 Research and Innovation Programmer (Grant No. 101001608). F.T.M. is supported by Generalitat Valenciana (Grant No. CIDEGENT/2018/041) and the Spanish Ministry of Science and Innovation (Grant Nos. PID2020-116578RB-I00 and EUR2022-134048). F.T.M. and M.D. are supported by the Marc R. Benioff Revocable Trust in collaboration with the World Economic Forum via the contract between ETH Zurich and University of Alicante “Mapping terrestrial ecosystem structure at the global scale.”

## Data availability

Data in the support of these findings and the R code for the statistical models are available from Figshare (https://doi.org/10.6084/m9.figshare.21879048).

## Supplementary Material

Supplementary_Information_wrae025
