# Supplementary material for: Stronger compensatory thermal adaptation of soil microbial respiration with higher substrate availability
Source: ISME J. 2024 Feb 12;18(1):wrae025. doi: 10.1093/ismejo/wrae025 (PMC10945366; doi:10.1093/ismejo/wrae025)
Supplement: Supplementary_Information_wrae025 [file supplementary_information_wrae025.pdf]

Supplementary Information for

Stronger compensatory thermal adaptation of soil microbial respiration with higher substrate availability

Lingrui Qu [a], Chao Wang [a,b] *, Stefano Manzoni [c], Marina Dacal [d,e], Fernando T. Maestre [d,f], Edith Bai [g,h]

[a] CAS Key Laboratory of Forest Ecology and Silviculture, Institute of Applied Ecology, Chinese Academy of Sciences, Shenyang, Liaoning, 110016, China

[b] Key Laboratory of Terrestrial Ecosystem Carbon Neutrality, Institute of Applied Ecology, Chinese Academy of Sciences, Shenyang, Liaoning, 110016, China

[c] Department of Physical Geography and Bolin Centre for Climate Research, Stockholm University, Stockholm, 10691, Sweden

[d] Instituto Multidisciplinar para el Estudio del Medio 'Ramón Margalef', Universidad de Alicante, Alicante, 03690, Spain

[e] Freie Universität Berlin, Institute of Biology, Berlin, 14195, Germany

[f] Departamento de Ecología, Universidad de Alicante, Alicante, 03690, Spain

[g] Key Laboratory of Geographical Processes and Ecological Security of Changbai Mountains, Ministry of Education, Northeast Normal University, Changchun, Jilin, 130024, China

[h] Key Laboratory of Vegetation Ecology, Ministry of Education, Northeast Normal University, Changchun, Jilin, 130024, China

***Corresponding author**

Chao Wang

Institute of Applied Ecology, Chinese Academy of Sciences

No. 72 Wenhua Road

Shenyang, Liaoning

110016, China
Telephone: +86-24-83970570
Email: cwang@iae.ac.cn

This file includes supplementary text, figure and tables.

Supporting text on models selection.

Fig. S1. Relationships between measured microbial respiration and mean annual temperature (MAT) at three incubation temperatures (T) without (a, c and e) and with substrate addition (b, d and f). (a) and (b) for the dataset from this study, (c) and (d) for the dataset for the dataset from Bradford *et al*. (Ref. 12), (e) and (f) for the dataset from Dacal *et al*. (Ref. 13).

Table S1. Geographical information, ecosystem types, climate and soil properties of the sampling sites.

Table S2. Unstandardized coefficients (mean ± s.d.), significance and R^2 values of predictive models used to assess the effect of temperature and pedoclimatic conditions on soil microbial respiration without substrate addition.

Table S3. Unstandardized coefficients (mean ± s.d.), significance and R^2 values of predictive models used to assess the effect of temperature and pedoclimatic conditions on soil microbial respiration with substrate addition.

Table S4. Standardized coefficients (mean ± s.d.), significance and R^2 values of predictive models used to assess the effect of temperature and pedoclimatic conditions on soil microbial respiration without substrate addition.

Table S5. Standardized coefficients (mean ± s.d.), significance and R^2 values of predictive models used to assess the effect of temperature and pedoclimatic conditions on soil microbial respiration with substrate addition.

Table S6. Parameters and R^2 values of the Macromolecular Rate Theory (MMRT) model.

Table S7. Parameters and R^2 values of the square-root model.

Table S8. Parameters and R^2 values of the Lloyd function.

Table S9. Standardized coefficients (mean ± s.d.), significance and R^2 values of predictive models used to assess the effect of temperature and pedoclimatic conditions on soil microbial respiration for datasets in Bradford *et al*. (Ref. 12) and Dacal *et al*. (Ref. 13).

Supporting text

Comparison of models describing the relationship between microbial respiration and temperature.

The most common approaches for measuring the response of soil microbial respiration to temperature have been the van's Hoff [1] and Arrhenius functions [2]. The former assumes that respiration scales as an exponential function of temperature (and is conceptually the same as the often-used Q_{10} model), while the later assumes that respiration scales as an exponential function of minus the inverse of temperature. Both the Arrhenius and Q_{10} models predict a monotonic increase in respiration rates as temperature rises, whereas both in the laboratory and in the field, a clearly identifiable temperature optimum in the unimodal trend of soil microbial respiration has been observed [3]. Thus, the Arrhenius model might limit extrapolation outside the optimal temperature range for microorganisms. Although the Q_{10} model has an advantage in its simplicity, it is not a constant value because it differs depending on the temperature interval used for calculation [4]. Hence, these two approaches may limit the scaling of the thermal responses of microbial respiration to warming. Over the past decades, many feasible alternative mathematical models have been used to improve theoretical understanding of thermal adaptation. For example, the square-root model developed by Ratkowsky *et al*. (Ref. 5) could effectively describe the direct effect of temperature on microbial respiration as well as the thermal adaptation of microorganisms. Subsequently, Lloyd and Taylor (Ref. 1) introduced a parameter into the Arrhenius function to modify the activation energy with temperatures, which has greatly improved the model's prediction. However, the square-root and Lloyd-functions cannot predict the decline in soil respiration above optimum temperatures. In this case, the macromolecular rate theory (MMRT) model, which considers mechanisms that may cause enzymatically driven nonlinearities with temperature, could better predict molecular responses to temperature [6-8]. Which of these different thermal response models should be selected, and how microbial adaptation would be included as their parameterization remain open questions.

We outline two criteria for evaluating model selection approaches: (1) giving a plausible result thermodynamically speaking and (2) providing a statistically superior fit [9]. We estimated the parameters of MMRT, square-root and Lloyd-function models for 11 sampling sites at three incubation temperatures with or without substrate addition, respectively (Supplementary Tables S6-S8). Furthermore, linear mixed-effect models include the most important drivers of microbial respiration to quantify thermal adaptation [10, 11].

We found that the MMRT model predicted thermodynamically plausible results for only ten out of 22 combinations (11 sampling sites × 2 substrate treatments, Supplementary Table S6). The square-root model predicted that the parameter $a \times T_{min}$ had statistically significant effects for only twelve out of 22 combinations, where T_{min} allowed for a direct descriptor of the thermal adaptation of microbial respiration (Supplementary Table S7). However, the Lloyd-function predicted statistically significant effects of β for 20 out of 22 combinations, where β was a parameter related to temperature sensitivity of microorganisms (Supplementary Table S8). We also found that results from the above three model approaches had similar R^2 regardless of the sampling sites and substrate treatment combinations. Thus, the Lloyd-function was selected for subsequent analyses due to its successful fits to data under substrate excess and depletion.

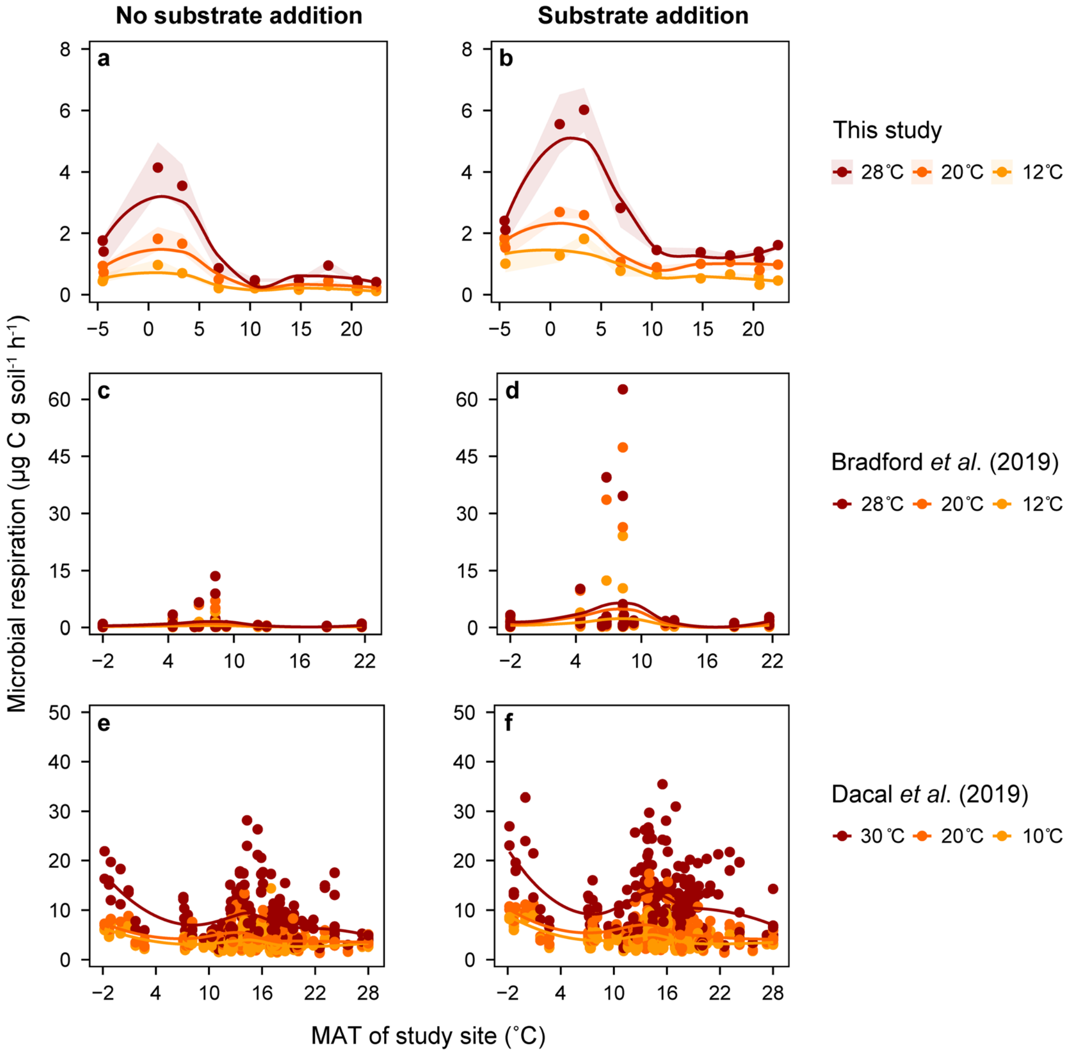

Fig. S1. Relationships between measured microbial respiration and mean annual temperature (MAT) at three incubation temperatures (T) without (a, c and e) and with substrate addition (b, d and f). (a) and (b) for the dataset from this study, **(c) and (d)** for the dataset for the dataset from Bradford *et al*. (Ref. 12), **(e) and (f)** for the dataset from Dacal *et al*. (Ref. 13).

Table S1. Geographical information, ecosystem types, climate and soil properties of the sampling sites.

Site name	Latitude (N°)	Longitude (E°)	Altitude (m)	MAP (mm)	MAT (°C)	TC (%)	TN (%)	pH	Clay (%)	Silt (%)	Sand (%)	Ecosystem types
MH	53.0	122.9	535.5	393.0	-4.4	5.40±1.68	0.23±0.06	5.52±0.09	49.45±3.46	28.03±0.65	22.53±2.99	Temperate forest
DXAL	51.5	123.2	805.0	356.8	-4.5	7.69±1.35	0.32±0.06	4.56±0.07	71.04±9.22	11.19±1.25	17.77±8.07	Temperate forest
WY	48.1	129.2	290.0	595.9	0.9	12.48±0.95	0.82±0.05	5.23±0.18	60.49±2.77	31.64±2.12	7.86±0.94	Temperate forest
CBS	42.4	128.1	784.0	610.9	3.3	11.96±1.16	1.02±0.10	5.09±0.12	47.46±2.05	35.68±1.66	16.86±1.71	Temperate forest
DLS	40.0	115.4	1179.8	513.3	6.9	5.78±0.78	0.49±0.06	6.70±0.12	35.01±1.76	28.90±2.30	36.09±2.82	Temperate forest
BTM	33.5	111.9	1402.0	713.1	10.5	3.83±0.40	0.30±0.03	4.96±0.09	47.23±0.97	26.32±2.37	26.45±2.62	Subtropical forest
JGS	31.9	114.1	197.0	903.3	14.8	2.85±0.32	0.22±0.02	4.22±0.02	27.77±1.39	14.18±1.84	58.04±1.47	Subtropical forest
WYS	28.1	117.7	630.0	1828.7	17.7	3.57±0.16	0.25±0.01	4.42±0.09	41.40±0.89	7.10±0.70	51.50±1.03	Subtropical forest
DHS	23.2	112.5	270.5	1743.3	20.6	3.74±0.45	0.26±0.03	3.74±0.10	58.47±3.78	12.99±3.08	28.54±6.28	Subtropical forest
JFL	18.7	108.9	860.0	1095.5	20.5	2.50±0.21	0.19±0.02	4.21±0.09	41.65±2.65	8.11±0.86	50.24±2.03	Tropical forest
XSBN	21.9	101.3	712.0	1528.9	22.4	2.50±0.19	0.22±0.01	4.18±0.04	55.69±3.19	21.85±1.99	22.46±1.73	Tropical forest

Abbreviations: MH, Mohe. DXAL, Daxinganling. WY, Wuying. CBS, Changbai Mountain. DLS, Dongling Mountain. BTM, Baotianman. JGS, Jigong Mountain. WYS, Wuyi Mountain. DHS, Dinghu Mountain. JFL, Jianfengling. XSBN, Xishuangbanna.

Table S2. Unstandardized coefficients (mean ± s.d.), significance and R^2 values of predictive models used to assess the effect of temperature and pedoclimatic conditions on soil microbial respiration without substrate addition.

Variables	Unstandardized Model (This study)				
	Model 1 (M1)	Model 2 (M2)	Model 3 (M3)	Model 4 (M4)	Model 5 (M5)
Intercept	**-2.919±0.533**	**-2.909±0.525**	**-3.225±0.613**	**-3.146±0.585**	**-3.237±0.244**
T	**0.077±0.005**	**0.077±0.005**	**0.077±0.005**	**0.077±0.005**	**0.077±0.005**
MAT	-0.018±0.011	-0.019±0.011	*-0.027±0.014*	*-0.027±0.014*	*-0.026±0.013*
SOC	**0.119±0.031**	**0.116±0.020**	NA	NA	NA
Biomass	**0.158±0.077**	**0.153±0.062**	**0.452±0.046**	**0.453±0.045**	**0.452±0.045**
TN	-0.075±0.588	NA	NA	NA	NA
DOC:DON	-0.014±0.015	-0.013±0.014	0.007±0.015	NA	NA
pH	-0.036±0.087	-0.040±0.081	-0.010±0.097	-0.017±0.096	NA
Clay	*0.005±0.003*	*0.005±0.003*	**0.013±0.003**	**0.013±0.003**	**0.013±0.003**
MAT × T	-0.0002±0.0003	-0.0002±0.0003	-0.0002±0.0004	-0.0002±0.0004	-0.0002±0.0004
Marginal R^2	0.87	0.87	0.82	0.82	0.83
Conditional R^2	0.92	0.92	0.92	0.92	0.92
AIC	151.23	150.02	170.04	161.68	156.84

Table S3. Unstandardized coefficients (mean ± s.d.), significance and R^2 values of predictive models used to assess the effect of temperature and pedoclimatic conditions on soil microbial respiration with substrate addition.

Variables	Unstandardized Model (This study)				
	Model 1 (M1)	Model 2 (M2)	Model 3 (M3)	Model 4 (M4)	Model 5 (M5)
Intercept	**-2.133±0.443**	**-2.100±0.441**	**-2.427±0.478**	**-2.126±0.446**	**-1.354±0.184**
T	**0.058±0.004**	**0.058±0.004**	**0.058±0.005**	**0.058±0.005**	**0.058±0.005**
MAT	-0.011±0.009	-0.013±0.009	*-0.019±0.010*	**-0.021±0.010**	**-0.029±0.009**
SOC	**0.108±0.028**	**0.094±0.018**	NA	NA	NA
Biomass	0.104±0.065	0.073±0.051	**0.291±0.034**	**0.284±0.034**	**0.275±0.032**
TN	-0.364±0.515	NA	NA	NA	NA
DOC:DON	0.004±0.013	0.008±0.012	*0.021±0.013*	NA	NA
pH	*0.124±0.071*	0.109±0.066	**0.159±0.072**	*0.132±0.070*	NA
Clay	-0.0004±0.0026	-0.0004±0.0027	**0.006±0.003**	**0.006±0.003**	*0.005±0.002*
MAT × T	0.0005±0.0003	0.0005±0.0003	0.0005±0.0003	0.0005±0.0003	0.0005±0.0003
Marginal R^2	0.83	0.82	0.80	0.80	0.80
Conditional R^2	0.87	0.87	0.85	0.85	0.85
AIC	118.33	117.32	135.37	129.29	127.14

Table S4. Standardized coefficients (mean ± s.d.), significance and R^2 values of predictive models used to assess the effect of temperature and pedoclimatic conditions on soil microbial respiration without substrate addition.

Variables	Standardized Model (This study)				
	Model 1 (M1)	Model 2 (M2)	Model 3 (M3)	Model 4 (M4)	Model 5 (M5)
Intercept	**-0.739±0.070**	**-0.739±0.071**	**-0.739±0.104**	**-0.739±0.103**	**-0.739±0.101**
T	**0.993±0.043**	**0.993±0.043**	**0.993±0.046**	**0.993±0.046**	**0.993±0.046**
MAT	**-0.414±0.177**	**-0.420±0.172**	**-0.575±0.236**	**-0.583±0.234**	**-0.567±0.208**
SOC	**0.917±0.241**	**0.893±0.157**	NA	NA	NA
Biomass	**0.426±0.208**	**0.412±0.167**	**1.216±0.123**	**1.220±0.122**	**1.218±0.120**
TN	-0.042±0.330	NA	NA	NA	NA
DOC:DON	-0.081±0.090	-0.076±0.081	0.040±0.089	NA	NA
pH	-0.059±0.141	-0.065±0.132	-0.017±0.158	-0.028±0.155	NA
Clay	*0.148±0.084*	*0.148±0.084*	**0.354±0.085**	**0.356±0.085**	**0.358±0.084**
MAT × T	-0.041±0.087	-0.041±0.087	-0.041±0.093	-0.041±0.093	-0.041±0.093
Marginal R^2	0.87	0.87	0.82	0.82	0.83
Conditional R^2	0.92	0.92	0.92	0.92	0.92
AIC	113.01	110.65	134.75	129.96	126.09

Table S5. Standardized coefficients (mean ± s.d.), significance and R^2 values of predictive models used to assess the effect of temperature and pedoclimatic conditions on soil microbial respiration with substrate addition.

Variables	Standardized Model (This study)				
	Model 1 (M1)	Model 2 (M2)	Model 3 (M3)	Model 4 (M4)	Model 5 (M5)
Intercept	**0.217±0.048**	**0.217±0.050**	**0.217±0.056**	**0.217±0.055**	**0.217±0.050**
T	**0.817±0.040**	**0.817±0.039**	**0.817±0.043**	**0.817±0.043**	**0.817±0.044**
MAT	-0.034±0.133	-0.058±0.130	-0.185±0.141	-0.227±0.138	**-0.368±0.107**
SOC	**0.833±0.212**	**0.723±0.135**	NA	NA	NA
Biomass	0.280±0.175	0.197±0.138	**0.784±0.093**	**0.765±0.092**	**0.741±0.087**
TN	-0.204±0.289	NA	NA	NA	NA
DOC:DON	0.024±0.078	0.048±0.070	*0.126±0.074*	NA	NA
pH	*0.201±0.115*	0.178±0.107	**0.258±0.117**	*0.214±0.114*	NA
Clay	-0.011±0.073	-0.011±0.074	**0.155±0.073**	**0.166±0.073**	*0.134±0.069*
MAT × T	0.123±0.079	0.123±0.079	0.123±0.086	0.123±0.086	0.123±0.087
Marginal R^2	0.83	0.82	0.80	0.80	0.80
Conditional R^2	0.87	0.87	0.85	0.85	0.85
AIC	80.11	77.95	100.08	97.56	96.39

Table S6. Parameters and R^2 values of the Macromolecular Rate Theory (MMRT) model [Ref. 6-8].

Site name	Substrate addition	MMRT model: $\ln(R_m) = \ln\left(\frac{k_B T}{h}\right) - \frac{\Delta H + \Delta C_p (T - T_0)}{RT} + \frac{\Delta S_{T_0} + \Delta C_p (\ln T - \ln T_0)}{R}$						
		ΔH	ΔS	ΔC_p	T_0	T_{opt}	T_{max}	R^2
MH	No	-44.14	-0.41	6.02	280	13.94	NA	0.20
DXAL	No	52.66	-0.07	0.50	280	-101.18	NA	0.73
WY	No	23.51	-0.16	3.01	280	-1.56	NA	0.71
CBS	No	59.01	-0.04	-0.37	315	212.39	149.12	0.78
DLS	No	9.16	-0.21	-2.31	315	47.12	29.00	0.57
BTM	No	66.03	-0.03	1.36	315	-8.17	NA	0.62
JGS	No	12.58	-0.21	-0.99	325	67.57	38.98	0.75
WYS	No	-21.42	-0.33	3.13	270	3.11	NA	0.78
DHS	No	30.30	-0.16	1.19	280	-20.23	NA	0.86
JFL	No	46.41	-0.10	0.68	280	-63.81	NA	0.84
XSBN	No	5.40	-0.23	-2.21	315	45.64	27.23	0.81
MH	Yes	2.37	-0.23	-1.79	310	39.78	19.82	0.33
DXAL	Yes	-15.02	-0.29	2.25	280	12.62	NA	0.26
WY	Yes	5.16	-0.20	-0.80	365	102.35	67.63	0.82
CBS	Yes	-19.05	**-0.31**	**5.40**	280	10.09	NA	0.89
DLS	Yes	-36.82	-0.38	7.21	280	11.78	NA	0.52
BTM	Yes	-16.01	-0.31	2.14	270	3.41	NA	0.84
JGS	Yes	16.07	**-0.19**	-3.32	300	32.61	18.04	0.90
WYS	Yes	**20.62**	**-0.17**	**-3.12**	295	29.41	14.57	0.90
DHS	Yes	**43.07**	**-0.10**	**-5.61**	295	30.13	18.89	0.94
JFL	Yes	**25.45**	**-0.16**	**-5.34**	295	27.23	15.84	0.86
XSBN	Yes	13.45	-0.19	-1.82	315	50.87	30.37	0.89

In the MMRT model, R_m is microbial respiration rate (μg C g soil^{-1} h^{-1}) at the temperature T (K), T_0 is reference temperature (K), k_B is Boltzman's constant (i.e., 1.3806 × 10^{-26} kJ K^{-1}), h is Planck's constant (i.e., 6.6261 × 10^{-37} kJ s), R is the universal gas constant (i.e., 8.314 × 10^{-3} kJ mol^{-1} K^{-1}), ΔH is the change in enthalpy (kJ mol^{-1}), ΔS is the change in entropy between the enzyme-substrate complex and the enzyme bound to the transition state at T_0 (kJ mol^{-1} K^{-1}), ΔCp is the change in the heat capacity of the enzyme (kJ mol^{-1} K^{-1}). T_{opt} corresponds to the temperature at maximum reaction rate (°C), T_{max} corresponds to the temperature where the rate of change is greatest (°C). We took the first and second derivative of MMRT to calculate T_{opt} and T_{max}, respectively. The T_0 value was set to approximately 10 °C below average temperature at the maximum rate. Significant ($P < 0.05$) parameters are shown in bold. The thermodynamically unreasonable results of the MMRT models were presented as $\Delta C_p > 0$ or $T_{opt} > 110$ °C.

Table S7. Parameters and R^2 values of the square-root model [Ref. 5].

Site name	Substrate addition	Square-root model: $\sqrt{R_m} = a \times (T - T_{min})$			
		a	$-a \times T_{min}$	T_{min}	R^2
MH	No	0.02	0.38	-19.00	0.19
DXAL	No	**0.04**	0.15	-3.75	0.75
WY	No	**0.06**	0.14	-2.33	0.67
CBS	No	**0.06**	0.05	-0.83	0.72
DLS	No	**0.03**	0.11	-3.67	0.55
BTM	No	**0.01**	**0.27**	-27.00	0.62
JGS	No	**0.02**	**0.19**	--9.50	0.74
WYS	No	**0.03**	0.18	-6.00	0.73
DHS	No	**0.02**	**0.16**	-8.00	0.86
JFL	No	**0.02**	0.10	-5.00	0.79
XSBN	No	**0.02**	**0.12**	-6.00	0.83
MH	Yes	**0.03**	**0.64**	-21.33	0.31
DXAL	Yes	0.02	**1.06**	-53.00	0.25
WY	Yes	**0.08**	0.18	-2.25	0.77
CBS	Yes	**0.07**	0.43	-6.14	0.80
DLS	Yes	**0.05**	0.19	-3.80	0.51
BTM	Yes	**0.02**	**0.50**	-25.00	0.82
JGS	Yes	**0.03**	**0.41**	-13.67	0.85
WYS	Yes	**0.02**	**0.59**	-29.50	0.86
DHS	Yes	**0.03**	**0.21**	-7.00	0.90
JFL	Yes	**0.03**	**0.48**	-16.00	0.78
XSBN	Yes	**0.04**	**0.23**	-5.75	0.88

In the square-root model, R_m is microbial respiration rate (μg C g soil^{-1} h^{-1}) measured at the temperature T (°C). T_{min} is the minimum temperature for microbial respiration (°C), and a is a slope parameter without any direct biological meaning, but related to the absolute microbial respiration rate. Significant ($P < 0.05$) parameters are shown in bold.

Table S8. Parameters and R^2 values of the Lloyd function [Ref. 1].

Site	Substrate	Lloyd equation: $\ln R_m = \ln\alpha + \beta T$		
name	addition	β	lnα	R^2
MH	No	0.05	**-1.43**	0.17
DXAL	No	**0.09**	**-1.88**	0.73
WY	No	**0.09**	**-1.22**	0.70
CBS	No	**0.10**	**-1.54**	0.77
DLS	No	**0.09**	**-2.67**	0.57
BTM	No	**0.05**	**-2.28**	0.62
JGS	No	**0.07**	**-2.61**	0.75
WYS	No	**0.07**	**-2.20**	0.77
DHS	No	**0.07**	**-2.82**	0.86
JFL	No	**0.08**	**-3.07**	0.84
XSBN	No	**0.08**	**-3.09**	0.80
MH	Yes	**0.05**	-0.71	0.33
DXAL	Yes	0.02	0.17	0.24
WY	Yes	**0.09**	**-0.89**	0.81
CBS	Yes	**0.07**	-0.38	0.85
DLS	Yes	**0.08**	**-1.47**	0.49
BTM	Yes	**0.05**	**-1.05**	0.83
JGS	Yes	**0.06**	**-1.29**	0.86
WYS	Yes	**0.04**	**-0.87**	0.84
DHS	Yes	**0.08**	**-2.02**	0.89
JFL	Yes	**0.06**	**-1.13**	0.78
XSBN	Yes	**0.08**	**-1.70**	0.89

In the Lloyd function, R_m is microbial respiration rate (μg C g soil^{-1} h^{-1}) measured at the temperature T (°C). α is basal respiration rate, and β represents the sensitivity to changes in temperature and is related to the commonly reported Q_{10} value. Significant ($P < 0.05$) parameters are shown in bold.

Table S9. Standardized coefficients (mean ± s.d.), significance and R^2 values of predictive models used to assess the effect of temperature and pedoclimatic conditions on soil microbial respiration for datasets in Bradford *et al*. (Ref. 12) and Dacal *et al*. (Ref. 13).

Variables	Bradford *et al*. (2019)		Dacal *et al*. (2019)	
	Control	Substrate addition	Control	Substrate addition
Intercept	**-1.217±0.060**	**-0.142±0.067**	**1.491±0.023**	**1.770±0.024**
T	**0.686±0.050**	**0.797±0.030**	**0.669±0.021**	**0.796±0.021**
MAT	**-0.333±0.124**	**-0.399±0.139**	**-0.296±0.046**	**-0.320±0.049**
Biomass	**1.244±0.147**	**1.467±0.164**	0.031±0.046	0.068±0.049
Texture	**-0.262±0.123**	*-0.267±0.138*	**-0.374±0.046**	**-0.493±0.049**
MAT × T	0.004±0.105	0.079±0.062	**-0.086±0.042**	0.029±0.042
Marginal R^2	0.58	0.65	0.50	0.57
Conditional R^2	0.83	0.95	0.78	0.83
AIC	247.19	135.61	531.56	545.95

Reference

1. Lloyd J, Taylor JA. On the temperature dependence of soil respiration. *Funct Ecol* 1994;**8**:315-23. https://doi.org/10.2307/2389824

2. Singh JS, Gupta SR. Plant decomposition and soil respiration in terrestrial ecosystems. *Bot Rev* 1977;**43**:449-528. https://doi.org/10.1007/BF02860844

3. Schipper LA, Hobbs JK, Rutledge S *et al*. Thermodynamic theory explains the temperature optima of soil microbial processes and high Q_{10} values at low temperatures. *Glob Chang Biol* 2014;**20**:3578-86. https://doi.org/10.1111/gcb.12596

4. Bååth E. Temperature sensitivity of soil microbial activity modeled by the square root equation as a unifying model to differentiate between direct temperature effects and microbial community adaptation. *Glob Chang Biol* 2018;**24**:2850-61. https://doi.org/10.1111/gcb.14285

5. Ratkowsky DA, Olley J, Mcmeekin TA *et al*. Relationship between temperature and growth rate of bacteria cultures. *J Bacteriol* 1982;**149**:1-5. https://doi.org/10.1128/jb.149.1.1-5.1982

6. Alster CJ, von Fischer JC, Allison SD *et al*. Embracing a new paradigm for temperature sensitivity of soil microbes. *Glob Chang Biol* 2020;**26**:3221-3229. https://doi.org/10.1111/gcb.15053

7. Alster CJ, Robinson JM, Arcus VL *et al*. Assessing thermal acclimation of soil microbial respiration using macromolecular rate theory. *Biogeochemistry* 2022;**158**:131-141. https://doi.org/10.1007/s10533-021-00885-6

8. Alster CJ, Baas P, Wallenstein MD *et al*. Temperature sensitivity as a microbial trait using parameters from macromolecular rate theory. *Front Microbiol* 2016;**7**:1821. https://doi.org/10.3389/fmicb.2016.01821

9. Alster CJ, Koyama A, Johnson NG *et al*. Temperature sensitivity of soil microbial communities: an application of macromolecular rate theory to microbial respiration. *J Geophys Res Biogeo* 2016;**121**:1420-1433. https://doi.org/10.1002/2016JG003343

10. Zhang Y, Li JT, Xu X *et al*. Temperature fluctuation promotes the thermal adaptation of soil microbial respiration. *Nat Ecol Evol* 2023;**7**:205-213. https://doi.org/10.1038/s41559-022-01944-3

11. Chen HY, Jing QF, Liu X *et al*. Microbial respiratory thermal adaptation is regulated by r-/K-strategy dominance. *Ecol Lett* 2022;**25**:2489-2499. https://doi.org/10.1111/ele.14106

12. Bradford MA, McCulley RL, Crowther TW *et al*. Cross-biome patterns in soil microbial respiration predictable from evolutionary theory on thermal adaptation. *Nat Ecol Evol* 2019;**3**:223-231. https://doi.org/10.1038/s41559-018-0771-4

13. Dacal M, Bradford MA, Plaza C *et al*. Soil microbial respiration adapts to ambient temperature in

global drylands. *Nat Ecol Evol* 2019;**3**:232-238. https://doi.org/10.1038/s41559-018-0770-5
